# Forecasting future dental health expenditures: Development of a framework using data from 32 OECD countries

**DOI:** 10.1111/cdoe.12597

**Published:** 2020-11-30

**Authors:** Milica Jevdjevic, Stefan Listl, Morgan Beeson, Maroeska Rovers, Yusuke Matsuyama

**Affiliations:** ^1^ Department of Dentistry ‐ Quality and Safety of Oral Healthcare Radboud University Medical Center Radboud Institute for Health Sciences Nijmegen The Netherlands; ^2^ Department of Conservative Dentistry Translational Health Economics Group Heidelberg University Heidelberg Germany; ^3^ School of Dental Sciences Newcastle University Newcastle upon Tyne UK; ^4^ Department of Operating Rooms Radboudumc Nijmegen The Netherlands; ^5^ Department of Health Evidence Radboudumc Nijmegen The Netherlands; ^6^ Graduate School of Medical and Dental Sciences Tokyo Medical and Dental University (TMDU) Global Health Promotion Bunkyo‐ku Japan

**Keywords:** dental diseases, dental healthcare costs, economic burden, economic forecasting

## Abstract

**Objective:**

To (1) develop a framework for forecasting future dental expenditures, using currently available information, and (2) identify relevant research and data gaps such that dental expenditure predictions can continuously be improved in the future.

**Methods:**

Our analyses focused on 32 OECD countries. Dependent on the number of predictors, we employed dynamic univariate and multivariate modelling approaches with various model specifications. For univariate modelling, an auto‐regressive (AR) dynamic model was employed to incorporate historical trends in dental expenditures. Multivariate modelling took account of historical trends, as well as of relationships between dental expenditures, dental morbidity, economic growth in terms of gross domestic product and demographic changes.

**Results:**

Estimates of dental expenditures varied substantially across different model specifications. Models relying on dental morbidity as one of the predictors performed worst regardless of their specification. Using the best‐fitted model specification, that is the univariate second‐order autoregression [AR(2)], the forecasted dental expenditures across 32 OECD countries amounted to US$316bn (95% forecasted interval, FI: 258‐387) in 2020, US$434bn (95%FI: 354‐532) in 2030 and US$594bn (95%FI: 485‐728) in 2040. Per capita spending in 2040 was forecasted to be highest in Germany (US$889, 95%FI: 726‐1090) and lowest in Mexico (US$52, 95%FI: 42‐64).

**Conclusions:**

The present study demonstrates the feasibility and challenges in predicting dental expenditures and can serve as a basis for improvement towards more sustainable and resilient health policy and resource planning. Within the limitations of available data sources, our findings suggest that dental expenditures in OECD countries could increase substantially over the next two decades and vary considerably across countries. For more accurate estimation and a better understanding of determinants of dental expenditures, more comprehensive data on dental spending and dental morbidity are urgently needed.

## 1 INTRODUCTION

The policy debate on health systems has been dominated in recent decades by concerns about growing cost pressures and nonsustainability of health systems' financing. If healthcare budgets are limited, increased expenditures for one type of care may mean that other types of care are no longer fundable to previous extents.^1^, ^2^ Hence, monitoring the dynamics of health expenditures is relevant to inform healthcare decision‐makers about potential challenges in the future financing of health care and to take timely action in the interest of population wellbeing. Up‐to‐date and reliable information on health expenditures is particularly important for decision‐makers who aim at allocating healthcare resources efficiently and equitably.^3^

Over recent decades, per capita global spending on health has almost doubled, from US$472 in 2000 to US$1007 in 2015.^4^ There has also been an increase in healthcare expenditures relative to the global economy: spending on health care amounted to 8.6% of the gross domestic product (GDP) in 2000, and it increased to 9.9% of GDP in 2015.^4^ Relying on historical trends, the Global Burden of Disease Health Financing Collaborator Network^5^ estimated that the global health expenditure will rise from US$9.21 trillion in 2014 to US$24.24 trillion in 2040. Against the background of increasing cost pressures in health care, such dynamics of rising health expenditures substantiate concerns about the sustainability of health systems.

Dental diseases are among the most prevalent and persistent diseases worldwide and impose a substantial economic burden to humankind.^6^, ^7^, ^8^, ^9^ Dental expenditures currently account for the third highest proportion of health spending in the European Union, with €90bn compared to €111bn spent on diabetes and €119bn spent on cardiovascular diseases.^9^, ^10^ Considerable proportions of dental expenditures are attributable to out‐of‐pocket payments in many countries. In addition to a greater financial hardship, future increases in dental expenditures could imply a substantial increase in unmet needs for dental care among less affluent populations. Dental expenditures are therefore particularly relevant to the United Nation (UN) and World Health Organization (WHO) goals of Universal Health Coverage.^11^, ^12^, ^13^

Despite recent attempts to better estimate the economic impacts of dental diseases, considerable room for improvement has been documented for the international reporting of dental expenditures, with mixed levels of availability of information across countries. The reporting of dental expenditures for member countries of the Organization for Economic Cooperation and Development (OECD) appeared to be more consistent than for several other countries.^7^, ^8^ Also, there is very little insight into the future economic implications of dental diseases, exceptions being work on dental expenditure in the US from The Centers for Medicare and Medicaid Services^14^ and by Nasseh, Vujicic.^15^ The aims of this study, therefore, are to (1) develop a framework for forecasting future dental expenditures, using currently available information, and (2) identify relevant research and data gaps such that dental expenditure predictions can continuously be improved in the future.

## 2 METHODS

For the development of the forecasting framework, we collated publicly available data on the predictors of interest (dental expenditure, morbidity, demographic changes and economic growth [GDP]). Dependent on the number of predictors, we employed both, dynamic univariate and multivariate modelling approaches with varying model specifications. The best‐performing model was identified based on performance indicators. Further details are provided below.

### 2.1 Data

Annual dental health expenditures (expressed as % of GDP) were derived from the OECD online platform—OECD Statistics (Health Expenditure and Financing) that relies on the Joint OECD, Eurostat and WHO National Health Accounts reports.^16^ Complete data were available for Denmark, Finland, Germany, Luxembourg, Switzerland, South Korea and the US. No dental expenditure data were available for Chile, Italy, Portugal and Turkey, and therefore, these countries were excluded from the analysis. For the remaining OECD countries, missingness of data ranged from 6% for Australia and Japan, to up to 76% for the United Kingdom and New Zealand (Table A1). Missing values on expenditure were imputed using the R package Amelia that utilizes a bootstrapped expectation‐maximization algorithm and has been commonly employed for multiple imputation of time‐series data.^17^ As a result, ten imputed datasets were created for further analysis. Absolute dental expenditures (in US$, adjusted for Purchasing Power Parity [PPP] to the 2010 price‐level) were calculated by multiplying dental expenditures (% of GDP) by country‐specific GDP^18^ for the respective year.

Disease estimates (incidence and prevalence of caries, periodontal disease and severe tooth loss) were obtained from the Global Burden of Disease database and were stratified by three age groups (persons younger than 15 years of age; persons age 15 or older to younger than 65 years of age; persons age 65 years or older) to account for demographic variations.^19^ Population sizes for the period 2000 to 2040 were derived from the United Nations database.^20^ Further details about the data sources used are shown in Table 1.

**Table 1:** Summary of variables and data sources

Variable	Year	Data source
Dental morbidity^a^	1990 to 2016	Institute for Health Metrics and Evaluation online database
Prevalence		
Caries in permanent teeth		
Periodontal disease		
Severe tooth loss		
Incidence		
Caries in permanent teeth		
Periodontal disease		
Severe tooth loss		
Population size^b^	2000 to 2040	United Nations database
Gross Domestic Product^c^	2000 to 2040	OECD database
Annual spending on dental health (percentage of GDP)	2000 to 2016^d^	OECD database

^a^ Stratified for three age groups (young: population aged less than 15 years; middle: population aged between 15 and 64 years; old: population older than 64 years).

^b^ Population aged up to 15 years; population aged between 15 and 64 years; population older than 64 years.

^c^ US $ PPP‐adjusted to 2010 values.

^d^ The frequency of reported information varied per country (see Appendix).

### 2.2 Forecasting morbidities

We estimated future dental morbidity (prevalence of dental diseases) for consideration as potential predictor variables in multivariate models.

Firstly, following the equation described in formula 1, the 2017 to 2040 incidence per 100 000 persons was forecasted for dental caries in permanent teeth, periodontal disease (ie, having a gingival pocket depth equal or more than 6 mm, or Community Periodontal Index of Treatment Needs score of 4, or a clinical attachment loss more than 6 mm), and severe tooth loss (ie, having less than nine remaining teeth). All variables were log‐scaled before deriving first difference.

(1) $$\text{Morbidity forecast formula}\;1:\Delta {\text{Incidence}}_{\text{c,y}}=\Delta {\text{Incidence}}_{c,y‐1}+\Delta {\text{Incidence}}_{c,y‐2}...+\Delta {\text{Incidence}}_{c,y‐n}+{\text{Country}}_{c}.$$

We built projection models for disease incidence using data for the period 1990‐2006 altering the number of auto‐regressive—[AR(i)] terms (ie, where i term denotes the number of lags of the dependent variable) with one or two lags. Namely, the change in incidence was regressed on itself lagged by one (a first‐order autoregression) or two periods (a second‐order autoregression). Thereby we estimated parameters forecasting future trends of dental morbidity. The model fitting was evaluated by their out‐of‐sample predictive validation with 2007‐2016 data by Root Mean Squared Error (RMSE)—the average of the square of the differences between predicted and original values. The model with the smallest RMSE was rerun using 1990‐2016 data, and the parameters were used to extrapolate the disease incidence through the year 2040. Then, as shown in formula 2, the same procedure was applied to forecast disease prevalence until 2040, using the forecasted disease incidence as an additional predictor.

(2) $$\text{Morbidity forecast formula}\;2:\Delta {\text{Prevalence}}_{c,y}=\Delta {\text{Incidence}}_{c,y}+\Delta {\text{Prevalence}}_{c,y‐1}+\Delta {\text{Prevalence}}_{c,y‐2}...\;+\Delta {\text{Prevalence}}_{c,y‐n}+\text{Country}.$$

where Δ: first difference, c: country, y: year, Country: country dummy variables.

### 2.3 Forecasting future dental health expenditure

To forecast future dental health expenditures, a dynamic modelling approach was applied as recommended by previous literature.^21^, ^22^ For the univariate modelling approach, we used existing and imputed dental health expenditure values from 2000 to 2016. In the multivariate approach and in addition to previous trends in dental health expenditures, dental morbidity, demographic changes and GDP were included as potential predictors.

The primary condition to produce reliable statistical inferences in time‐series analysis is data stationarity.^23^ The statistical properties (variance, mean, autocorrelation) of stationary time‐series are constant and do not change over time. The Augmented Dickey‐Fuller test was performed, and in agreement with previous studies,^21^ data stationarity was confirmed.

We performed linear regression analyses using time‐series data of the first differences (ie, a change in variable value within a period of one year) of log‐transformed variables. In brief, we applied four types of models: Expenditure forecast model 1 is a univariate model relying exclusively on previous trends in dental health expenditure as predictor variable, whereas expenditure forecast models 2‐4 are multivariate and rely on different sets of multiple predictors.

$$\text{Expenditure forecast model}\;1:\Delta {\text{DHE}}_{c,y}=\Delta {\text{DHE}}_{c,y-1}+\Delta {\text{DHE}}_{c,y-2}\ldots +\Delta {\text{DHE}}_{c,y-n}+{\text{Country}}_{c}.$$

$$\text{Expenditure forecast model}\;2:\Delta {\text{DHE}}_{c,y}=\Delta {\text{DHE}}_{c,y-1}+\Delta {\text{DHE}}_{c,y-2}\ldots +\Delta {\text{DHE}}_{c,y-n}+\Delta {\text{Prevalence}}_{c,a,d,y-1}+\Delta {\text{Pop}}_{c,a,y-1}+\Delta {\text{GDP}}_{c,y-1}+{\text{Country}}_{c}.$$

$$\text{Expenditure forecast model}\;3:\Delta {\text{DHE}}_{c,y}=\Delta {\text{DHE}}_{c,y-1}+\Delta {\text{DHE}}_{c,y-2}\ldots +\Delta {\text{DHE}}_{c,y-n}+\Delta {\text{Pop}}_{c,a,y-1}+\Delta {\text{GDP}}_{c,y-1}+{\text{Country}}_{c}.$$

$$\text{Expenditure forecast model}\;4:\Delta {\text{DHE}}_{c,y}=\Delta {\text{DHE}}_{c,y-1}+\Delta {\text{DHE}}_{c,y-2}\ldots +\Delta {\text{DHE}}_{c,y-n}+\Delta {\text{Prevalence}}_{c,a,d,y-1}+\Delta {\text{GDP}}_{c,y-1}+{\text{Country}}_{c}.$$

where Δ: first difference, DHE: dental health expenditure, Country: country dummy variables, Prevalence: the prevalence of dental diseases, Pop: the size of population, GDP: gross domestic product, the subscripts c, y, a and d, indicate country, year, age group (<15 years‐old, 15‐64 years‐old and ≥ 65 years‐old) and dental diseases (caries, periodontitis, and severe tooth loss), respectively. Note that all variables were log‐scaled before deriving first differences.

Similar to morbidity predictions (see above), we ran models using data covering the period 2000‐2011 and changing the number of AR terms with one or two lags. Model fit was assessed by means of the out‐of‐sample predictive validation in relation to 2012‐2016 data and using RMSE. We reran the model with the smallest RMSE using 2000‐2016 data to obtain parameters for the extrapolation up until the year 2040. Forecast performance of the models was assessed using RMSE and Mean Absolute Percentage Error (MAPE) as performance indicators. MAPE represents the average absolute per cent error for each time period minus actual values divided by actual values.

### 2.4 Forecast uncertainty

To consider the uncertainty for the forecasting models, we estimated the 95% forecasted intervals (FIs), assuming that the model uncertainty is constant for the period of 2017‐2040. The lower and upper bounds of the 95% forecasted intervals (FI) of the first difference (D1) were estimated by D1(n) = D1 (±) 1.96 × RMSE, where RMSE was based on the difference between the predicted values and observed values between 2012‐2016.

### 2.5 Auxiliary analyses

Auxiliary analyses were conducted to assess the potential implications of additional dental care systems characteristics. In addition to population needs (expressed as morbidity, see above) and the number of practicing dentists per 1000 people, dental care utilization was captured by the number of yearly dental visits per capita. Further details are provided in the Appendix.

## 3 RESULTS

Table 2 reports the performance of the various forecasting models. According to the RMSE and MAPE criterions, the best‐performing model was yielded by the univariate second‐order autoregression [AR(2)]. Among the multivariate models considered, a specification according to formula 5 performed best. Models relying on dental morbidity as one of the predictors performed worst regardless of the number of lags in their specification.

**Table 2:** Comparison of forecast performance of four best‐performing models across different variable specifications using Root Mean Squared Error (RMSE) and Mean Absolute Percentage Error (MAPE) as indicators using in‐sample data 2012‐2016

Model	RMSE	MAPE
Model 1 UniVar (DHE)	0.0842	464.39
Model 2 MultiVar (DHE, Morbidity, GDP, POP)	0.0869	527.83
Model 3 MultiVar (DHE, GDP, POP)	0.0846	485.94
Model 4 MultiVar (DHE, Morbidity, GDP)	0.0867	496.23

Note

Variable used for forecasting: DHE, dental health expenditure; Morbidity‐Prevalence of dental diseases; GDP, gross domestic product; POP, population size.

Abbreviations: UniVar, Univariate approach; MultiVar, Multivariate approach.

Table 3 shows baseline and forecasted OECD countries’ spending on dental health across different models. The dental expenditures for the 32 examined OECD countries amounted to US$247.2bn in 2015 (baseline year). Forecasted dental expenditures ranged between US$307.7bn and US$331.6bn in 2020, US$407.2bn and US$472.2bn in 2030, and US$536.7bn and US$712.3bn in 2040.

**Table 3:** OECD: Dental expenditures 2015‐2040 across different model specifications, US$ adjusted for PPP to the 2010 price‐level, billion

	2015	2020	2030	2040
Model 1 UniVar (DHE)	247.16 (baseline year)	316.48 (258.29‐387.78)	434.18 (354.35‐532.00)	594.41 (485.12‐728.32)
Model 2 MultiVar (DHE, Morbidity, GDP, POP)		320.62 (260.13‐395.18)	439.79 (356.82‐542.06)	590.25 (478.90‐727.50)
Model 3 MultiVar (DHE, GDP, POP)		331.62 (269.32‐408.32)	472.17 (391.43‐593.46)	712.33 (578.51‐877.11)
Model 4 MultiVar (DHE, Morbidity, GDP)		307.74 (250.00‐378.82)	407.16 (330.77‐501.21)	536.71 (436.00‐660.67)

Note

Variable used for forecasting: DHE, dental health expenditure; Morbidity‐Prevalence of dental diseases; GDP, gross domestic product; POP, population size.

Abbreviations: UniVar, Univariate approach; MultiVar, Multivariate approach.

Based on the forecasts yielded by the best‐performing model (Model 1), spending on dental health was projected to increase from US$247.2bn spent in 2015 to US$316.5bn (95%FI: 258.3‐387.8) in 2020, US$434.2bn (95%FI:354.4‐532.0) in 2030 and US$594.4bn (95%FI: 485.1‐728.3) in 2040 (Table 3). In per capita terms, on average, OECD spending on dental health will grow from US$221 spent in 2015 to US$277 (95%FI: 226‐339) in 2020, US$365 (95%FI: 298‐447) in 2030 and US$487 (95%FI: 397‐597) in 2040.

As displayed in Table 4, aggregate country‐level expenditures in 2015 were the highest for the United States (US$109.4bn), Germany (US$29.5bn) and Japan (US$25.6bn). Our estimates, based on predictions out of the best‐performing model, suggest a similar trend for the future, with the same highest‐spending countries across the period 2015 to 2040. The United States is projected to spend the highest amount with US$143.4bn (95%FI: 117.1‐ 175.7) in 2020, $198.0bn (95%FI: 161.6‐242.6) in 2030 and $272.8bn (95%FI: 222.6‐334.2) in 2040. The lowest aggregate country‐level dental spending is found for Iceland with forecasts of $92.9 million (95%FI: 75.8‐113.8) in 2020, $123.41 million (95%FI: 100.7‐151.2) in 2030 and $164.0 (95%FI: 133.8‐200.9) in 2040.

**Table 4:** Dental health expenditure from 2015‐2040, based on predictions out of the best‐performing model (total, US$ adjusted for PPP to the 2010 price‐level, billion)

Country	Expenditure 2015	Expenditure 2020	Expenditure 2030	Expenditure 2040
Australia	5.80	6.38 (5.20‐7.81)	8.69 (7.09‐10.65)	11.79 (9.69‐14.45)
Austria	1.86	2.38 (1.94‐2.91)	3.22 (2.63‐3.94)	4.34 (3.54‐5.32)
Belgium	1.83	2.09 (1.70‐2.56)	2.81 (2.30.3.45)	3.80 (3.10‐4.65)
Canada	10.66	13.05 (10.65‐15.99)	17.78 (14.51‐21.78)	24.20 (19.75‐29.65)
Czech Republic	1.20	1.40 (1.15‐1.72)	1.89 (1.54‐2.32)	2.55 (2.08‐3.12)
Denmark	1.41	1.87 (1.52‐2.29)	2.52 (2.06‐3.09)	3.40 (2.78‐4.17)
Estonia	0.17	0.17 (0.14‐0.21)	0.23 (0.19‐0.28)	0.31 (0.25‐0.38)
Finland	0.56	0.63 (0.51‐0.77)	0.84 (0.69‐1.03)	1.13 (0.92‐1.38)
France	11.76	15.73 (12.82‐19.25)	21.46 (17.53‐26.30)	29.25 (23.87‐35.84)
Germany	29.47	38.41 (31.36‐47.06)	52.69 (43.00‐64‐56)	72.12 (58.86‐88.37)
Greece	1.03	1.90 (1.55‐2.33)	2.59 (2.11‐3.17)	3.49 (2.85‐4.27)
Hungary	0.76	0.87 (0.71‐1.06)	1.16 (0.95‐1.42)	1.56 (1.27‐1.91)
Iceland	0.08	0.09 (0.08‐0.11)	0.12 (0.10‐0.15)	0.16 (0.13‐0.20)
Ireland	0.59	0.91 (0.74‐1.11)	1.22 (1.00‐1.50)	1.64 (1.34‐2.02)
Israel	1.19	1.55 (1.26‐1.89)	2.09 (1.70‐2.55)	2.81 (2.29‐3.44)
Japan	25.61	32.40 (26.44‐39.69)	44.50 (36.32‐54.53)	60.86 (49.67‐74.58)
Latvia	0.11	0.12 (0.10‐0.14)	0.16 (0.13‐0.19)	0.21 (0.17‐0.25)
Lithuania	0.25	0.19 (0.15‐0.23)	0.25 (0.25‐0.30)	0.33 (0.27‐0.41)
Luxembourg	0.14	0.17 (0.14‐0.21)	0.23 (0.19‐0.28)	0.30 (0.25‐0.37)
Mexico	3.55	4.46 (3.64‐5.46)	6.04 (4.93‐7.41)	8.19 (6.68‐10.03)5
Netherlands	3.42	4.27 (3.49‐5.24)	5.79 (4.73‐7.10)	7.84 (6.40‐9.61)
New Zealand	0.62	0.45 (0.36‐0.55)	0.59 (0.48‐0.73)	0.80 (0.65‐0.97)
Norway	1.57	1.87 (1.52‐2.29)	2.52 (2.05‐3.08)	3.39 (2.77‐4.16)
Poland	2.44	2.74 (2.24‐3.36)	3.71 (3.02‐4.54)	5.01 (4.09‐6.13)
Slovakia	0.51	0.48 (0.39‐0.58)	0.64 (0.52‐0.78)	0.85 (0.70‐1.04)
Slovenia	0.15	0.22 (0.18‐0.27)	0.29 (0.24‐0.36)	0.39 (0.32‐0.47)
South Korea	6.65	4.52 (3.68‐5.53)	6.04 (4.93‐7.40)	8.18 (6.68‐10.03)2
Spain	10.19	12.28 (10.02‐15.04)	16.73 (13.66‐20.50)	22.77 (18.59‐27.90)
Sweden	2.63	2.94 (2.40‐3.60)	3.97 (3.24‐4.87)	5.37 (4.38‐6.58)
Switzerland	2.81	3.58 (2.92‐4.38)	4.85 (3.96‐5.94)	6.56 (5.35‐8.03)
United Kingdom	8.78	15.00 (12.25‐18.38)	20.57 (16.79‐25.20)	28.02 (22.07‐34.34)
United States	109.37	143.43 (117.06‐175.74)	197.99 (161.59‐242.60)	272.79 (222.63‐334.24)

**Figure 1:**
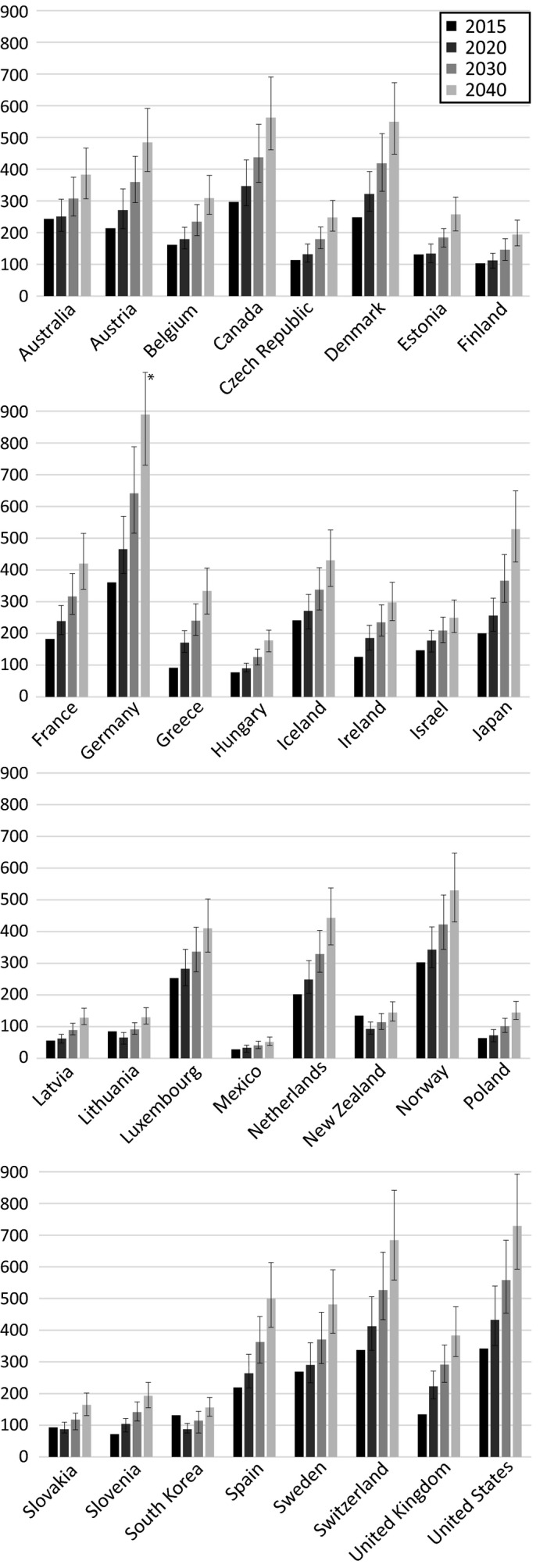
Country‐level spending on dental health from 2015‐2040, based on predictions out of the best‐performing model (per capita, US$ adjusted for PPP to the 2010 price‐level). *For better readability the maximum scale value was fixed at 900; the exact 2040 estimate for Germany is $889 (95%FI: 726, 1090)

Figure 1 illustrates country‐specific per capita spending on dental health (adjusted for PPP to the 2010 price‐level). There are substantial country‐level variations for the period 2015‐2040. Per capita spending on dental health is depicted to grow for all OECD countries (2015‐2040) except Lithuania, New Zealand, Slovakia and South Korea. The highest per capita expenditures in 2040 were forecasted for Germany (US$889 [95%FI: 726‐1090]), followed by the United States (US$729 [95%FI: 595‐896]), Switzerland (US$684 [95%FI: 558‐838]), Canada (US$563 [95%FI: 459‐690]) and Denmark (US$550 [95%FI: 449‐673]) (see Table A2 for detailed numeric estimates). By contrast, Mexico had the lowest predicted dental expenditures per capita in 2040 (US$52 [95%FI: 42‐64]), ranking lower than Latvia (US$129; 95%FI: 105‐157) and Lithuania (US$129; 95%FI: 106‐159).

The auxiliary analyses revealed that additional factors such as utilization of dental services (eg. number of yearly dental consultations per capita) and density of dental providers (eg. number of practicing dentists per 1000) might also be important to account for when quantifying future dental expenditure (see Appendix). Regression analyses indicated that the density of dental providers is significantly associated with the amount of dental expenditure in countries with lower economic growth (GDP per capita lower than the OECD mean). However, the extent to which additional analysis could be performed was restricted by nonavailable or incomplete data.

## 4 DISCUSSION

Our findings highlight substantial uncertainties in forecasting future dental expenditures on basis of currently available data and large variations across different specifications of prediction models. Notwithstanding, on basis of the best‐performing model, dental expenditures in 32 OECD countries are predicted to rise substantially, with an aggregated spending estimated to range between US$485.12bn and US$728.32bn in 2040. In addition, the findings highlight considerable cross‐country variations in per capita dental expenditures, ranging from US$52 in Mexico to US$889 in Germany in 2040.

To our knowledge, this is the first study that provides a systematically developed framework for predicting future dental expenditures up until the year 2040 and facilitates cross‐country comparisons. In addition to the historical trend in dental expenditure, we incorporated the most recent updates on the trends in dental morbidity, economic growth and demographic changes as potential predictors for future spending on dental care. In line with previous literature on economic forecasting,^21^, ^22^ these factors were hypothesized to capture population needs and demand for dental health care. In contrast to our expectations, all models considered within the multivariate approach failed to perform better than an AR(2) based on RMSE and MAPE scores.

The extent and quality of currently available public data on variables of interest posed particular methodological challenges. Firstly, even with targeting only the OECD countries, data on dental expenditures were consistently reported only for seven (Denmark, Finland, Germany, Switzerland, Luxembourg, South Korea, the United States) out of 36 OECD countries. Having in mind that the longitudinal datasets on important dental healthcare factors were not identifiable even for developed countries, there is an urgent need to gather reliable and relevant information in more systematic and transparent ways. Secondly, due to the absence of comparable high quality national data on dental morbidity, we used the publicly available estimates of the Global Burden of Disease Study.^19^ Consequently, this might be a reason that the multivariate models relying on the morbidity trends were not among the better‐performing.

Finally, in economic forecasting of health expenditures, the level of data aggregation determines the magnitude of correlation between potential predictors and healthcare costs.^24^ When aggregated on a macro‐level, as it was the case in our study, the magnitude of association between health expenditure and income/economic growth surpasses the one with health status/morbidity. With smaller units of observation, however, the effects of policy measures on a micro‐level can be evaluated and taken into account. This is particularly important to answer ‘what if’ questions when cost‐containment measures are considered (lowering the share of publicly‐financed dental services, increase in co‐payments, reducing the coverage in the insurance package, etc). For example, a recent study^25^ has shown that price liberalization can change composition of dental care utilization resulting in a decrease in demand for preventive services due to higher out‐of‐pocket payments. We could not differentiate between preventive services, cosmetic or restorative care. While debatable, it was assumed that population needs may also partly be reflected in dental care utilization. An auxiliary analysis was performed to examine the effect of reported utilization in terms of the amount of dental visits. However, within the limitations of available data, no statistically significant association could be detected. In the future and upon availability of more granular data, it may be possible to better elucidate the determinants of dental expenditures and patterns of dental services consumption across different population groups.

The main limitation of the analyses was that time‐series data follow relatively continuous trends whereas changes in health and nonhealth sectors, such as policy reforms, economic disruption or technological innovation can have a significant impact on health expenditure. As a result, possible evolvement in dental care provision was not captured by the forecasting models. As several studies have highlighted, role substitution^26^, ^27^ and different remuneration systems^28^, ^29^ could alter future trajectories of dental health care financing. Moreover, the number of (dental) healthcare providers^30^, ^31^ may be an important driver of health expenditures, as corroborated by our auxiliary analysis. In addition, potential implications of unpredictable events like the recent COVID‐19 outbreak which has caused suspension of dental care cannot be ruled out. This may result in significant changes in dental spending, as suggested by Nasseh, Vujicic.^32^ All the more, forecasts of dental expenditures are highly important to closely monitor and predict the dynamics of dental expenditure to equip oral health systems when dealing with the consequences of unanticipated events (external shocks).

For a more robust assessment of the future economic trajectories, additional inputs such as the share of out‐of‐pocket payments, private insurance coverage and government spending on dental health are necessary. Countries with greater spending on dental health do not automatically have better oral health outcomes or financial risk protection.^33^ Therefore, economic forecasting can also be a useful tool to compare and improve the performance of oral healthcare systems. Moreover, the pay‐off of resources dedicated to the current preventive services could be evaluated and provide a guidance for future investments in oral health care.

Our findings revealed promising new avenues for future research. With investing in better data collection, processing and predictive modelling, policy makers would be able to anticipate future needs and identify gaps with available resources. This is particularly relevant at the time of the increasing support to integrate oral health into universal health coverage.^34^ The present study substantiates both the societal relevance and the methodological challenges involved in providing robust and reliable predictions of future dental expenditures. Health systems and resource planning could benefit from these findings, as they emphasize the critical importance of more comprehensive health economic monitoring as a key information source for sustainable and resilient health policy and resource allocation.

## 5 CONCLUSION

In conclusion, our findings show that given the expected future dynamics of dental expenditures and continuing uncertainties in oral health systems planning (including due to unexpected events such as the COVID‐19 pandemic), coordinated health policy action is needed to attenuate the predicted economic burden and to warrant efficiency and sustainability of dental care systems.

## CONFLICT OF INTEREST

The authors did not receive any financial support and declare no potential conflicts of interest with respect to the publication of this manuscript.

## AUTHOR CONTRIBUTIONS

MJ contributed to conception, design, data acquisition and interpretation, performed analyses, drafted and critically revised the manuscript. SL contributed to conception, design, interpretation and critically revised the manuscript. MB contributed to interpretation, performed analyses and critically revised the manuscript. MR contributed to interpretation and critically revised the manuscript. YM contributed to conception, design, interpretation, performed analyses and critically revised the manuscript. All authors gave their final approval and agreed to be accountable for all aspects of the work.

## Data Availability

The data that support the findings of this study are available from the corresponding author upon reasonable request.

## Appendix

Forecasting future dental health expenditures: Development of a framework using data from 32 OECD countries

Milica Jevdjevic, Stefan Listl, Morgan Beeson, Maroeska Rovers, Yusuke Matsuyama

## AUXILIARY ANALYSES

We have searched for information on additional factors that could potentially drive dental health expenditures (eg, utilization, the percentage of the population with a yearly dental visit, the number of dentists per population, out‐of‐pocket payments). We have found a high rate of discrepancy in data availability between the countries. Data on the number of yearly dental consultations per capita and the number of dentists per 1000 people were the most consistent.^16^ However, information on dental utilization was completely missing for Iceland, Israel, Latvia, Norway, Slovenia and Switzerland. On the other hand, data on providers density was not available for Greece, Ireland, New Zealand, Slovak Republic, South Korea, Spain and the United States. Taking into account already limited data on dental expenditures, the scarcity of information on all variables of interest across multiple time‐points deterred more comprehensive analysis. On all available observations, a multiple linear regression was performed to explore the correlation between per capita dental expenditures, GDP per capita, dental utilization as well as the dentist density. As shown in Table A1, when applied on the entire sample only GDP per capita appeared to be a significant determinant of dental health expenditures (β = 1.147, *P* = .000), whereas dental utilization (β = 0.084, *P* = .245) and dentist density (β = 0.252, *P* = .067) were not significant. If the analysis was performed exclusively on the countries whose GDP per capita was lower than the OECD mean in the respective year, in addition to economic growth (GDP per capita; β = 1.668, *P* = .000), dentist density was positively correlated with dental expenditures (β = 0.817, *P* = .007) while dental utilization remained nonsignificant (β = 0.031, *P* = .780). However, that was not the case among the countries with GDP per capita higher than the OECD mean for which utilization shown the strongest relationship, although not statistically significant.

**Table A1:** Results of the multiple linear regression analyses of the determinants associated with dental health expenditure per capita

	All countries	Countries with GDP per capita^a^ <OECD mean	Countries with GDP per capita^b^ >OECD mean
β (GDP per capita) β (dental utilization) β (dentist density)	1.147 (*P* = .000) 0.084 (*P* = .245) 0.252 (*P* = .067)	1.668 (*P* = .000) 0.031 (*P* = .780) 0.817 (*P* = .007)	0.216 (*P* = .082) −0.132 (*P* = .399) 0.154 (*P* = .556)
R‐squared	0.9806	0.8962	0.9745
Number of observations	205	69	87

^a^ Estonia, Hungary, Lithuania, Mexico, Poland.

^b^ Germany, Luxembourg, Netherlands, Austria, Denmark, Finland.

**Table A2:** Information on annual dental health expenditure per OECD country

Country	First year reported	Latest year reported	No. of years reported	Percentage of missing information
Australia	2000	2015	16	5.9%
Canada	2003	2016	14	17.7%
Czech Republic	2003	2016	14	17.7%
Denmark	2000	2016	17	0.0%
Estonia	2003	2016	14	17.7%
Finland	2000	2016	17	0.0%
France	2003	2016	14	17.7%
Germany	2000	2016	17	0.0%
Greece	2009	2016	8	52.9%
Hungary	2003	2016	14	17.7%
Iceland	2003	2016	14	17.7%
Ireland	2011	2016	6	64.7%
Israel	2006	2014	9	47.0%
Japan	2000	2015	16	5.9%
South Korea	2000	2016	17	0.0%
Latvia	2004	2016	13	23.5%
Lithuania	2004	2016	13	23.5%
Luxembourg	2000	2016	17	0.0%
Mexico	2003	2016	14	17.7%
Netherlands	2003	2016	14	17.7%
New Zealand	2004	2007	4	76.4%
Norway	2003	2016	14	17.7%
Poland	2003	2016	14	17.7%
Slovakia	2005	2016	12	29.4%
Slovenia	2003	2016	14	17.7%
Spain	2003	2016	14	17.7%
Sweden	2011	2016	6	64.7%
Switzerland	2000	2016	17	0.0%
United Kingdom	2013	2016	4	76.4%
United States	2000	2016	17	0.0%

**Table A3:** OECD ranking; Dental health expenditure from 2015‐2040, 95%FI, per capita, Int.‐$ (2010)

Germany	361	Germany	465 (380, 570)	Germany	641 (523, 786)	Germany	889 (726, 1090)
United States	342	United States	433 (353, 530)	United States	558 (456, 684)	United States	729 (595, 894)
Switzerland	337	Switzerland	412 (337, 505)	Switzerland	527 (430, 645)	Switzerland	684 (558, 838)
Norway	303	Canada	347 (283, 425)	Canada	438 (357, 536)	Canada	563 (459, 690)
Canada	297	Norway	343 (280, 420)	Norway	422 (345, 518)	Denmark	550 (448, 673)
Sweden	269	Denmark	322 (263, 395)	Denmark	419 (342, 513)	Norway	530 (432, 649)
Luxembourg	253	Sweden	291 (237, 356)	Sweden	371 (303, 454)	Japan	528 (431, 647)
Denmark	248	Luxembourg	282 (230, 346)	Japan	366 (299, 448)	Spain	500 (408, 613)
Australia	244	Austria	271 (221, 332)	Spain	363 (296, 445)	Austria	485 (395, 594)
Iceland	241	Iceland	271 (221, 332)	Austria	360 (293, 441)	Sweden	481 (393, 590)
Spain	220	Spain	264 (216, 324)	Iceland	338 (275, 414)	Netherlands	443 (362, 543)
Austria	214	Japan	256 (209, 341)	Luxembourg	337 (275, 413)	Iceland	430 (351, 527)
Netherlands	202	Australia	251 (205, 308)	Netherlands	329 (269, 403)	France	420 (343, 515)
Japan	200	Netherlands	249 (203, 305)	France	316 (258, 387)	Luxembourg	410 (335, 503)
France	182	France	239 (195, 293)	Australia	308 (251, 377)	Australia	383 (313, 470)
Belgium	162	United Kingdom	223 (182, 273)	United Kingdom	291 (238, 357)	United Kingdom	383 (313, 470)
Israel	147	Ireland	185 (151, 227)	Greece	240 (196, 294)	Greece	334 (272, 409)
New Zealand	135	Belgium	180 (147, 220)	Ireland	234 (191, 287)	Belgium	309 (252, 378)
United Kingdom	134	Israel	177 (145, 217)	Belgium	234 (191, 287)	Ireland	297 (243, 364)
South Korea	131	Greece	171 (140, 210)	Israel	209 (170, 256)	Estonia	258 (210, 316)
Estonia	131	Estonia	134 (109, 164)	Estonia	185 (151, 227)	Israel	249 (203, 305)
Ireland	126	Czech Republic	132 (108, 162)	Czech Republic	180 (147, 220)	Czech Republic	248 (202, 304)
Czech Republic	113	Finland	112 (92, 137)	Finland	146 (120, 179)	Finland	194 (158, 238)
Finland	103	Slovenia	104 (85, 128)	Slovenia	141 (115, 173)	Slovenia	193 (158, 237)
Slovakia	93	New Zealand	92 (75, 113)	Hungary	125 (102, 154)	Hungary	178 (145, 218)
Greece	92	Hungary	90 (73, 110)	Slovakia	118 (96, 145)	Slovakia	164 (134, 201)
Lithuania	85	South Korea	88 (72, 107)	South Korea	115 (94, 140)	South Korea	156 (127, 191)
Hungary	78	Slovakia	87 (71, 107)	New Zealand	114 (93, 140)	New Zealand	145 (118, 177)
Slovenia	72	Poland	72 (59, 89)	Poland	101 (83, 124)	Poland	144 (118, 177)
Poland	64	Lithuania	66 (54, 81)	Lithuania	91 (75, 112)	Lithuania	129 (106, 159)
Latvia	56	Latvia	62 (51, 76)	Latvia	89 (73, 110)	Latvia	128 (105, 157)
Mexico	28	Mexico	33 (27, 41)	Mexico	41 (33, 50)	Mexico	52 (42, 64)
